# Rare coronary artery anomaly: left anterior descending artery origin form right coronary cusp

**DOI:** 10.1093/omcr/omae015

**Published:** 2024-03-25

**Authors:** Tomohiro Nakajima, Keitaro Nakanishi, Tsuyoshi Shibata, Keishi Ogura, Nobuyoshi Kawaharada

**Affiliations:** Department of Cardiovascular Surgery, Sapporo Medical University School of Medicine, Hokkaido, Sapporo, Japan; Department of Cardiovascular Surgery, Sapporo Medical University School of Medicine, Hokkaido, Sapporo, Japan; Department of Cardiovascular Surgery, Sapporo Medical University School of Medicine, Hokkaido, Sapporo, Japan; Division of Radiology and Nuclear Medicine, Sapporo Medical University Hospital, Hokkaido, Sapporo, Japan; Department of Cardiovascular Surgery, Sapporo Medical University School of Medicine, Hokkaido, Sapporo, Japan

## INTRODUCTION

Coronary artery anomalies are reported to be less than 1%. In this case, we report an incidentally discovered coronary artery anomaly during preoperative assessment for abdominal aortic aneurysm surgery [1].

## CASE REPORT

**Figure 1:**
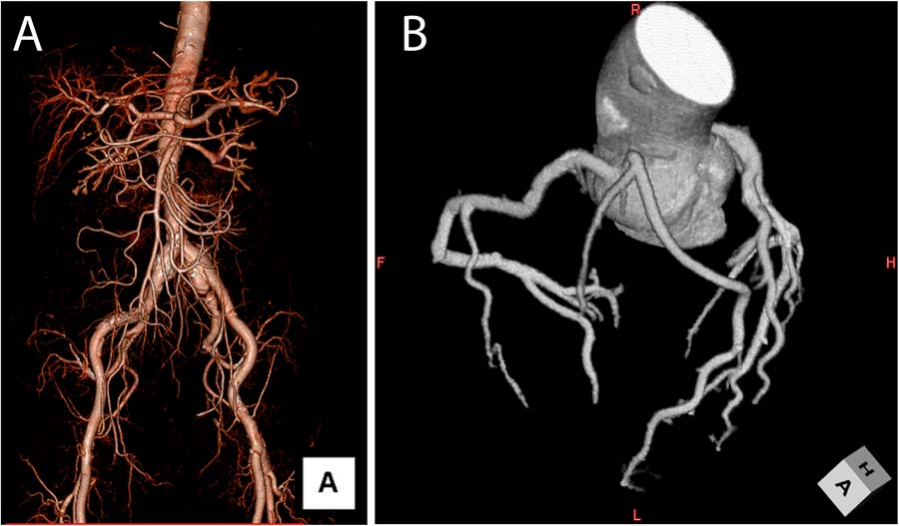
Volume rendering computed tomography. (**A**) Bilateral common iliac arteries were found, with a diameter of 30 mm. (**B**) Coronary Volume rendering CT. The anterior descending branch of the left coronary artery originated from the right coronary apex cusp, and the vessel originating from the left coronary apex cusp perfused the left diagonal and circumflex regions.

The patient was a 77-year-old man. He was referred to our hospital because of bilateral common iliac artery aneurysms of 30 mm in diameter (Fig. 1A). He had never been diagnosed with any heart disease or abnormality during medical examinations. When he underwent coronary angiography 3D-CT as a preoperative evaluation for aneurysm surgery, it was found that he had an arterial malformation in which the anterior descending branch of the left coronary artery was protruding from the cusp of the right coronary apex. The left diagonal and circumflex branches of the left coronary artery ran from the cusp of the left coronary apex (Fig. 1B). The frequency of anomalous coronary artery originating from the opposite sinus of Valsalva with prepulmonic course is reported to be 0.04% [2].

According to reports from various authors, perioperative myocardial ischemia may occur during abdominal aortic aneurysm surgery with coronary artery lesions. In this case, the patient was treated with EVAR (Excluder) and discharged on the sixth postoperative day without any postoperative problems.

We have a case report of a left coronary artery aneurysm associated with an abdominal aortic aneurysm. The frequency of this abnormality was 0.04%. Although there was no coronary event, this information is very useful in the event of a coronary event in the future [3]. It is advisable to examine the coronary arteries during the preoperative examination for abdominal aortic aneurysm.

## DISCUSSION

We encountered a case of coronary artery anomaly found during preoperative evaluation for abdominal aortic aneurysm. This case was particularly rare among coronary artery anomalies.
